# F204. THE DANISH HIGH-RISK AND RESILIENCE STUDY - VIA 7 - A PROSPECTIVE COHORTE STUDY OF 522 7 YEARS OLD CHILDREN BORN TO PARENTS DIAGNOSED WITH SCHIZOPHRENIA OR BIPOLAR DISORDER - RESULTS ON PSYCHOPATHOLOGY, COGNITION AND LIVING CONDITIONS

**DOI:** 10.1093/schbul/sby017.735

**Published:** 2018-04-01

**Authors:** Anne Amalie Thorup, Nicoline Hemager, Ditte V Ellersgaard, Camilla Jerlang Christiani, Birgitte Klee Burton, Katrine S Spang, Maja Gregersen, Anne Søndergaard, Ditte L Gantriis, Aja Greve, Jens Richardt Jepsen, Ole Mors, Kerstin von Plessen, Merete Nordentoft

**Affiliations:** 1 Child and Adolescent Mental Health Center Capital Region of Denmark; 2 Mental Health Center, Capital Region of Denmark, The Lundbeck Foundation Initiative for Integrative Psychiatric Research (iPSYCH); 3 Aarhus University Hospital, The Lundbeck Foundation Initiative for Integrative Psychiatric Research (iPSYCH); 4 Center for Neuropsychiatric Schizophrenia Research, Child and Adolescent Mental Health Center, Mental Health Services, Capital Region of Denmark; 5 University Hospital Lausanne

## Abstract

**Background:**

For decades familial high-risk studies have informed us about genetic and environmental risk factors for schizophrenia and recently also bipolar disorder. Familial high-risk studies are important and relevant and may represent a possible shortcut to learning more about early markers of illness, mental vulnerability and resilience.

**Methods:**

The Danish High Risk and Resilience Study – VIA 7 is a prospective cohort study of 522 7-year old children, 202 of them born to at least one parent diagnosed with schizophrenia in the Danish registries, 120 of them born to a least one parent diagnosed with bipolar disorder and 200 of them born to parents without any of these diagnoses. A comprehensive battery has been used combining assessments from several domains for both parents and children.

**Results:**

Results show that children born to parents with schizophrenia or bipolar disorder have higher frequencies of early mental problems. Further there are marked differences between the three groups concerning neuro cognition, motor functioning and living conditions including socioeconomic status, early risk factors and home environment - all factors that are known to be important with regard to healthy child development.

**Discussion:**

First results from the VIA 7-study indicate that many children and families have unmet needs and problems. Perspectives are two-fold: we aim to follow the cohort and conduct a new assessment before puberty (at age 11). Simultaneously, we are evolving an early, integrated, specialized and family based intervention, called VIA Family, to prevent or ameliorate development of severe mental illness in individuals born to parents with schizophrenia or bipolar disorder.

